# Smith‐Lemli‐Opitz syndrome — Fetal phenotypes with special reference to the syndrome‐specific internal malformation pattern

**DOI:** 10.1002/bdr2.1620

**Published:** 2019-12-16

**Authors:** Katharina Schoner, Martina Witsch‐Baumgartner, Jana Behunova, Robert Petrovic, Rainer Bald, Susanne G. Kircher, Annette Ramaswamy, Britta Kluge, Matthias Meyer‐Wittkopf, Ralf Schmitz, Barbara Fritz, Johannes Zschocke, Franco Laccone, Helga Rehder

**Affiliations:** ^1^ Institute of Pathology Philipps‐University Marburg Marburg Germany; ^2^ Institute of Human Genetics Medical University Innsbruck Innsbruck Austria; ^3^ Institute of Medical Genetics Medical University Vienna Vienna Austria; ^4^ Institute of Medical Biology Comenius University Bratislava Bratislava Slovakia; ^5^ Clinic of Gynecology and Obstetrics Klinikum Leverkusen Leverkusen Germany; ^6^ Clinic of Gynecology and Obstetrics University Clinic Oldenburg Oldenburg Germany; ^7^ Clinic of Gynecology and Obstetrics University Clinic Muenster Münster Germany; ^8^ Institute of Human Genetics Philipps‐University Marburg Marburg Germany

**Keywords:** atrioventricular septal defect, bilateral renal agenesis, DHCR7 gene mutations, fetal Smith‐Lemli‐Opitz syndrome, holoprosencephaly

## Abstract

**Background:**

Autosomal‐recessive SLOS is caused by mutations in the *DHCR7* gene. It is defined as a highly variable complex of microcephaly with intellectual disability, characteristic facies, hypospadias, and polysyndactyly. Syndrome diagnosis is often missed at prenatal ultrasound and fetal autopsy

**Methods:**

We performed autopsies and *DHCR7* gene analyses in eight fetuses suspected of having SLOS and measured cholesterol values in long‐term formalin‐fixed tissues of an additional museum exhibit

**Results:**

Five of the nine fetuses presented classical features of SLOS, including four cases with atrial/atrioventricular septal defects and renal anomalies, and one with additional bilateral renal agenesis and a Dandy‐Walker cyst. These cases allowed for diagnosis at autopsy and subsequent SLOS diagnosis in two siblings. Two fetuses were mildly affected and two fetuses showed additional holoprosencephaly. These four cases and the exhibit had escaped diagnosis at autopsy. The case with bilateral renal agenesis presented a novel combination of a null allele and a putative C‐terminus missense mutation in the *DHCR7* gene

**Conclusions:**

In view of the discrepancy between the prevalence of SLOS among newborns and the carrier frequency of a heterozygous *DHCR7* gene mutation, the syndrome‐specific internal malformation pattern may be helpful not to miss SLOS diagnosis in fetuses at prenatal ultrasound and fetal autopsy

## 1 INTRODUCTION

Smith‐Lemli‐Opitz syndrome (SLOS OMIM #270400) is an autosomal recessive metabolic disorder affecting the last step of cholesterol synthesis. The syndrome was first described in 1964 (Smith, Lemli, & Opitz, 1964). In 1993 low cholesterol and high 7‐dehydrocholesterol levels were found in SLOS patients, indicating a possible 7‐dehydrocholesterolreductase (7‐DHCR) deficiency (Tint et al., 1994). Consequently, causative mutations were identified in the *DHCR7* gene (Fitzky et al.1998; Moebius, Fitzky, Lee, Paik, & Glossmann, 1998; Wassif et al., 1998; Waterham et al., 1998). To date 218 variants of the *DHCR7* gene (HGMD professional, June 2019) have been described; the c.964‐1G > C (former name IVS8‐1G > C) splice site mutation in intron 8 is by far the most common mutant (null) allele in SLOS (Waterham & Hennekam, 2012; Witsch‐Baumgartner et al., 2000; Wright et al., 2003). Major features involve intrauterine growth retardation; microcephaly; characteristic facies with bitemporal narrowing, short nose, anteverted nostrils, long philtrum, microgenia, and low set ears; postaxial hexadactyly (more frequent on hands); syndactyly of Toes 2–3; genital anomalies (in males ranging from cryptorchidism and microphallus to hypospadias and ambiguous genitalia [pseudovaginal perineoscrotal hypospadias], in females less notable), hypotonia, and various degrees of intellectual disability. Associated malformations include cleft palate, septal defects of the heart, renal and cerebral anomalies; the latter comprise ventricular dilatation, defects of the corpus callosum and cerebellar vermis, and in severely affected cases, holoprosencephaly (HPE) (Kelley & Hennekam, 2000).

Cholesterol deficiency causes deficiency of its metabolite dihydrotestosterone, thus being responsible for the genital anomalies in affected males. Cholesterol binds to the morphogen sonic hedgehog (SHH) and acts as a modifier of the signaling cascade involved in patterning of the limbs, heart, and midline structures of the brain. HPE and other malformations in SLOS are thought to result from incomplete or abnormal modification of the SHH protein due to cholesterol deficiency (Kelley et al., 1996). Prenatal diagnosis can be performed on the biochemical or—more reliably—on the DNA level (Loeffler, Utermann, & Witsch‐Baumgartner, 2002; Mills et al., 1996). Preimplantation genetic diagnosis has also been reported (Liss, Lukaszuk, Bruszczynska, Szczerkowska, & Rebala, 2008).

The incidence of SLOS varies with populations; SLOS is relatively frequent in Caucasians (Canada 1:22,700), particularly in Slavs (Czech Republic 1:10,000) and rare in persons of African and Asian origin. Compared to the prevalence rate the carrier frequencies of a *DHCR7* mutation were unexpectedly high, namely 1:51 among 9,109 Americans from Northwestern Europe, 1:235 among 3,284 Americans of African ancestry, and 1:67 among 51,237 Americans of all populations (Bzdúch, Behulová, & Skodová, 2000; Lazarin, Haque, Evans, & Goldberg, 2017; Nowaczyk et al., 2001; Witsch‐Baumgartner et al., 2008; Wright et al., 2003). Currently there is no treatment proven permanently effective for patients with SLOS. The benefits of dietary cholesterol supplementation and of simvastattin therapy are controversially discussed (Porter, 2008).

We report on nine fetuses with SLOS, in which the diagnosis was confirmed by molecular *DHCR7*‐ and/or biochemical analysis and discuss the diagnostic relevance of the fetal malformation pattern for prenatal diagnosis. As a curiosity, we show an exhibit with SLOS from the Patho‐anatomical Collection in the Fool's Tower in Vienna.

## 2 METHODS

Nine fetuses were included after positive prenatal testing by targeted biochemical analysis performed because of an affected sibling, or after biochemical and molecular confirmation of clinical post abortion or postnatal SLOS diagnosis in the presence of characteristic SLOS features. In addition to these nine cases, highly suspective of having SLOS, all our other chromosomally normal three cases with holoprosencephaly, and hexadaytyly as well as all our other 11 unclassified cases with ambiguous genitalia (two also with hexadactyly or toe II/III syndactyly, respectively) were tested without identification of a *DHCR7* mutation and were thus excluded. Eight fetuses had been sent for autopsy and syndrome diagnosis to the “Working Group of Fetal Pathology and Syndromology” at the Institute of Medical Genetics (1990–2003) and at the Institute of Pathology (2004–2019) in Marburg from different obstetric departments in Germany. The additional exhibit of the Fool's Tower Collection in Vienna was transferred to Marburg for completion of autopsy. Autopsy included thorough photographic documentation, determination of adapted severity scores (see footnote b in Table 1) (Kelley & Hennekam, 2000), X‐ray, cytogenetic and histological examinations (for technical details see Schoner et al., 2017). In Cases 1, 4, and 6, the fetal bodies were incomplete due to artifactual lesions or fragmentation during termination of pregnancy or because of a preceding incomplete autopsy in 1966 with removal of abdominal organs only; an autopsy report of that time was not available. The exhibit had been stored in 10% formaldehyde since 1966. Skin biopsies including subcutaneous tissues were taken and biochemical cholesterol analysis from Case 6 and a phenotypically normal control specimen of comparable age and fixation was performed by gas chromatography—mass spectrometry at the Institute of Medical Biology in Bratislava, Slovakia. Briefly, cholesterol and other neutral sterols were saponified, then extracted with hexane and derivatized with BSTFA (bistrimethylsilyltrifluoroacetamid) according to Kelley (1995).

**Table 1:** Clinical and molecular findings

Clinical, autopsy and laboratory data	Case 1	Case 2	Case 3	Case 4	Case 5	Case 6	Case 7	Case 8	Case 9
Parental ethnicity	German	German	German	German	Turkish (non‐consang.)	Presumably Austrian	Mat. Mazedonian/pat. German	German	German
Maternal age	36 years	39years	36 years	30 years	25 years	ND	23 years	25 years	35 years
Gravida/para	III/II	II/I	IV/III	IV/0	IV/I	ND	II/I	III/I	V/II
Previous losses and/or child with malformations	1 boy—2years Mild SLOS (cholest. 68 mg/dl)	1 boy (dec)—SLOS	1 boy (dec)—Polydactyly Intersex, AVCD	2 early losses + 1 termination Malformations	2 early losses	ND	−	1 tubal pregn.	1 tubal pregn. + Case 8
Prenatal ultrasound	Normal	VSD Hypoplast. Genit. *Recurr. SLOS*	Hexadactyly intersex AVSD *Recurr. MMS*	Hexadactyly syndactyly intersex *VATER assoc*.	CLP Brachymelia, Fem. Genitalia *Campom. Dysplas. (SOX9‐Mut.Excl.)*	ND	Anhydramnion hygroma, AVSD hexadactyly diGeorge syndrome	ND	HPE—*Trisomy 13 suspected and excluded*
Prenatal chromos. Analysis	*46,XX*	*46,XY*	*46,XY*	*46,XY[50]/47,XY,+mar[3]*	*46,XY*	ND (*female*)	*46,XY*	*46,XY*	*46,XY*
Gest. Week at termination	12th	15th	23rd	20th	22nd	Preterm	18th	35th	36th
Fetal weight/fetal crown‐heel length IUGR confirmed	4,2 g/5 cm (3,5 cm CRL) ?	28 g/12,5 cm (9 cm CRL) ?	390 g (< third p^a^)/29 cm +	135 g (< third p^a^)/18 cm+	285 g (< third p^a^)/25,5 cm	? /46 cm = < third p ?	149 g/19,5 cm (13 cm CRL) ?	1850 g (< third p^a^)/30 cm +	2,430 g (< 10th p^a^)/47 cm +
Characteristic facies	−	−	+	+	+	+	+	Cebocephaly with PMA	Cebocephaly with PMA
Polydactyly of hands right/left	—/—	—/—	+^5^/+^5^	—/+^5^	—/—	+^6^/+^6^	+^5^/+^5^	+^6^/+^6^	+^5^/—
Polydactyly of feet right/left	—/—	—/—	+^5^/+^5^	+^6^/—^5^	—^4^/—^6^	—/—	—/—	—/—	—/—
Syndactyly of second and third toe	—/—	+/+	+/+	+/+	+/+	+/+	+/+	+/+	+/+
Heart defect	−	−	AVSD	ASD1 + 2 (single atrium)	−	ASD2	AVSD	ASD2 VAoS	AVSD VAoA
Renal anomaly—right/left	Ectopic/normal	Horseshoe kidney	Ectopic/Hypoplastic	Artifactual defects	Agenesis/double kidney	ND	Agenesis/Agenesis	Hypoplastic/normal	Ectopic /micro‐cysts
Hypospadias	Female	PSH	PPSH	PPSH	PPSH	Female	PPSH	PPSH	PPSH
Lung hypoplasia/defective lobulation	—/—	—/+	+/+	+/+	+/+	+/+	+/+	+/+	+/+
Major brain anomalies	−	−	−	−	−	DWC	DWC	HPE semilobar	HPE alobar
Other	−	Mobile cecum	Brachycolon, SD	Artifactual defects	CLP, mobile cecum, SD	SD	Bifid uvula/epi‐glottis, cataract, SD	Microph‐thalmia, Colobomas SD	Microphthalmia
Severity score^b^	5	15	45	>30	45	>35	60	70	70
Pre‐(post‐)natal biochem. Analysis	Cholesterol ↓ 7‐ u. 8‐DHC↑	Cholesterol ↓ 7‐ u. 8‐DHC ↑	ND	ND	ND	Cholesterol ↓↓↓	ND	ND	ND
*DHC‐*mutation	p.Val326Leu/p.Arg352Trp	p.Gly410Ser/p.Leu99Pro	IVS8‐1 G > C/IVS8‐1 G > C	IVS8‐1 G > C/IVS8‐1 G > C	IVS8‐1 G > C/IVS8‐1 G > C	ND	p.Trp151Ter/p.Cys451Arg	IVS8‐1 G > C IVS8‐1 G > C	IVS8‐1 G > C IVS8‐1 G > C

*Note: −* ^*4*^ */+* ^*6*^ = numbers indicate numbers of metacarpals in the absence (−) or presence (+) of hexadactyly IVS8‐1G > C = c.964‐1G > C (p.Gly322LysfsX136).

Abbreviations: ASD, atrial septal defect; AVSD, atrio‐ventricular septal defect; CLP, cleft lip/palate; CRL, crown‐rump length; dec, deceased; DWC, Dandy‐Walker cyst; HPE, holoprosencepaly; IUGR, intrauterine growth retardation; MMS, multiple malformation syndrome; MS, mitral valve stenosis; ND, not determined; PMA, premaxillary agenesis; PPSH, pseudovaginal perineoscrotal hypospadias; PSH, penoscrotal hypospadias; SD, sacral dimple; VAoA/VAoS, valvular aortic atresia/stenosis.

a Fetal weight charts (Nicolaides, Wright, Syngelaki, Wright, & Akolekar, 2018). Percentiles of fetal lengths were not considered because of brachymelia. There are no fetal weight charts published below 20 weeks.

b Severity score for SLOS = Sum of scores (one point for milder malformations, two points for more severe malformations in relation to 10 organ systems) with the maximal point number of 20 taken as 100% (Kelley & Hennekam, 2000). In the present study premaxillary agenesis is scored as two points in the oral organ system equating it with median cleft lip due to premaxillary agenesis. Renal hypoplasia, representing the most common renal anomaly, is scored with one point in the renal organ system regarding it as a mild renal anomaly, comparable to renal ectopia or cortical cysts.

Molecular analyses were performed after informed consent was obtained from the parents, excluding Case 6. The tests were carried out at the Institutes of Human Genetics in Innsbruck, Heidelberg and Vienna. Genomic DNA from living family members and from the deceased affected fetuses was extracted from blood samples and frozen tissues (Cases 1 to 5 and 7) according to standard procedures. The coding exons and adjacent intronic sequences of the *DHCR7* gene (transcript reference sequence: LRG_340, NM_001360.2) were amplified by polymerase chain reaction (PCR) with previously reported flanking primers and were analyzed by Sanger sequencing on ABI Genetic Analyzers 310 and 3,730 (Fitzky et al., 1998).

## 3 RESULTS

Clinical data, prenatal ultrasound, autopsy and laboratory findings are summarized in Table 1.

### 3.1 Prenatal SLOS diagnosis

Prenatal SLOS diagnosis was made in Cases 1 and 2 by biochemical analysis on chorionic villus samples. The analysis had been performed due to an affected sibling with proven SLOS. Thus, low cholesterol and elevated 7‐ and 8‐DHC levels were detected, proving the recurrence of SLOS. The fetuses represented “*mild manifestations*” of SLOS, documented by a severity score of 5 to 15.

### 3.2 Post‐abortion clinical diagnosis of SLOS

**Figure 1:**
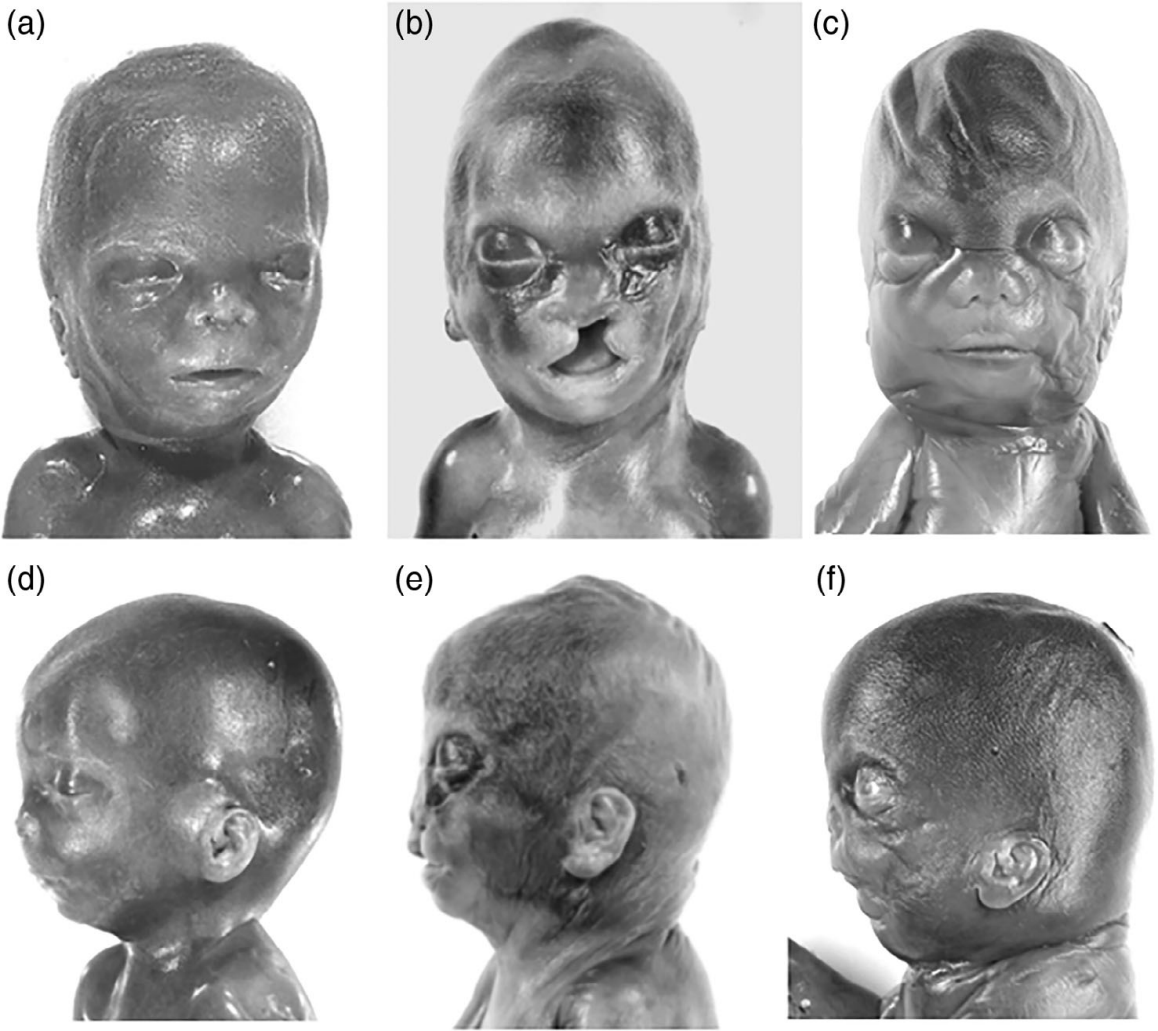
Facial aspects and profiles of fetal SLOS in Case 3 at 23 weeks (a, d) Case 5 at 22 weeks (b, e), and Case 7 at 18 weeks (c, f). The male fetuses display prominent front with bitemporal narrowing, hypertelorism, short upturned nose, low set, dorsally rotated ears, long smooth philtrum, narrow lips, and microretrognathia. In Case 5, additional unilateral cleft lip and palate, and in Case 7 hydrops in the presence of bilateral renal agenesis were observed. SLOS, Smith‐Lemli‐Opitz syndrome

Post‐abortion clinical diagnosis of SLOS was made in Cases 3 to 5 and 7 at fetal autopsy based on the presence of recognizable SLOS features. Most of these features had already been seen on prenatal ultrasound, but were not classified as part of a SLO syndrome. In addition to polydactyly, toe II/III syndactyly, hypospadias, cardiac, renal, and cerebral anomalies (Table 1), classical SLOS features in these fetuses included intrauterine growth retardation (IUGR), brachymelia, and brachydactyly. Craniofacial dysmorphia comprised a prominent occiput and a flat face with bitemporal narrowing, hypertelorism, short flat upturned nose, long smooth philtrum, microretrognathia, and low set, dorsally rotated ears (Figure 1a–f). The severity score in Cases 3 to 5 ranged from >30 to 45, thus corresponding to a “*more severe classical manifestation*” of the disorder, whereas Case 7, presenting with bilateral renal agenesis and a severity score of 60, had to be classified as a “*severe and lethal manifestation”* of SLOS.

### 3.3 Retrospective SLOS diagnosis

**Figure 2:**
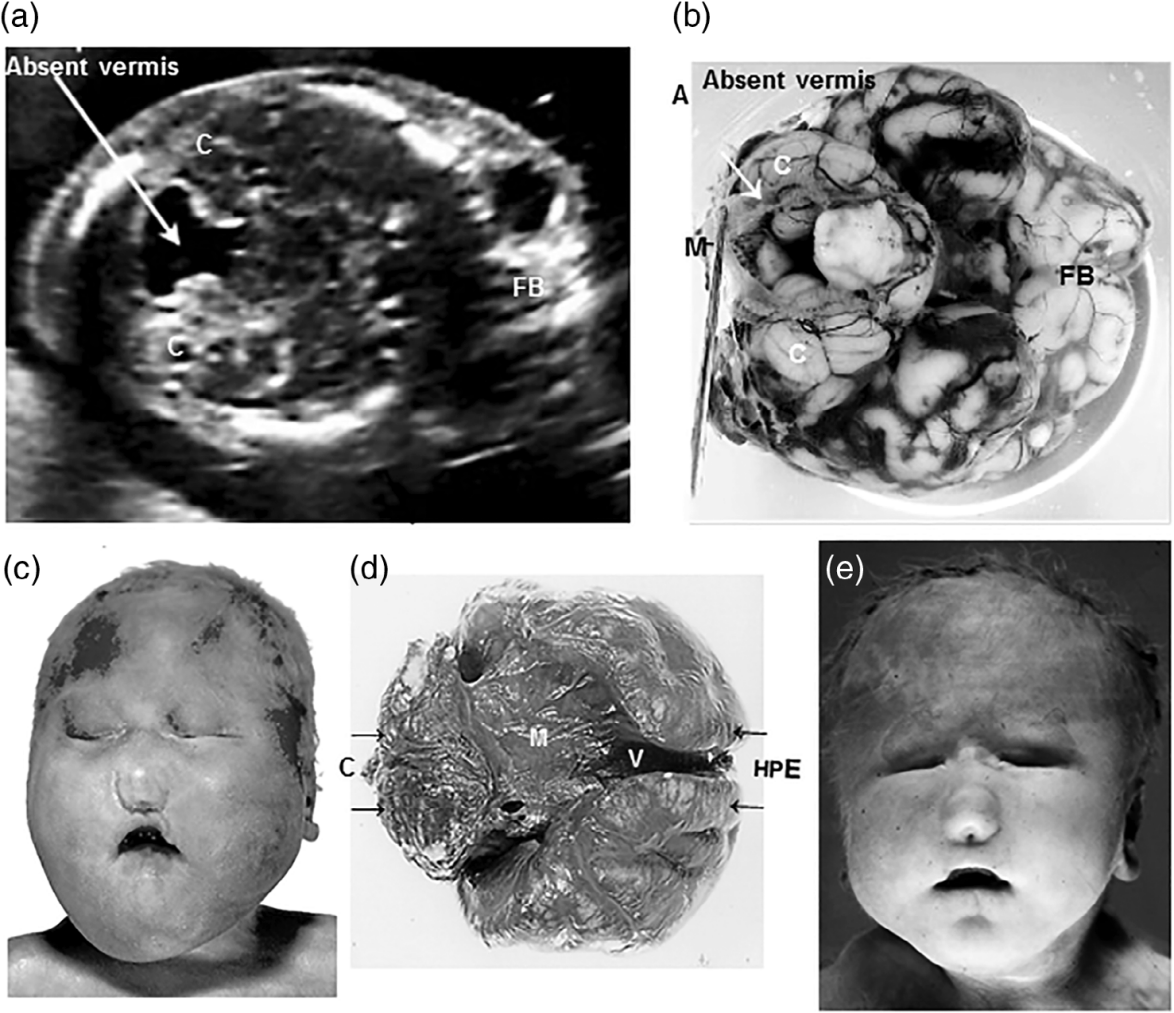
Agenesis of the cerebellar vermis with the remaining roofing membrane of the fourth ventricle distended to form a Dandy‐Walker cyst (DWC) in Case 7 as shown by prenatal ultrasound (a) and on a basal view of the brain at autopsy with the ruptured DWC on the left indicated by a lying probe (b). Facial aspects of the male siblings in Case 8 at 35 weeks (c) and Case 9 at 36 weeks (e) display cebocephaly with hypotelorism and a small nose with a single nostril. An occipital view of the brain of Case 8 shows lobar holoprosencephaly with a horseshoe‐shaped fused forebrain and dilatation of common cerebral ventricles, roofed by a collapsing membrane (d). FB, forebrain; HPE, holoprosencephalic forebrain; C, cerebellum; M, membrane, covering the DWC in Case 7 (b) and the common ventricular system in Case 8 (d)

**Figure 3:**
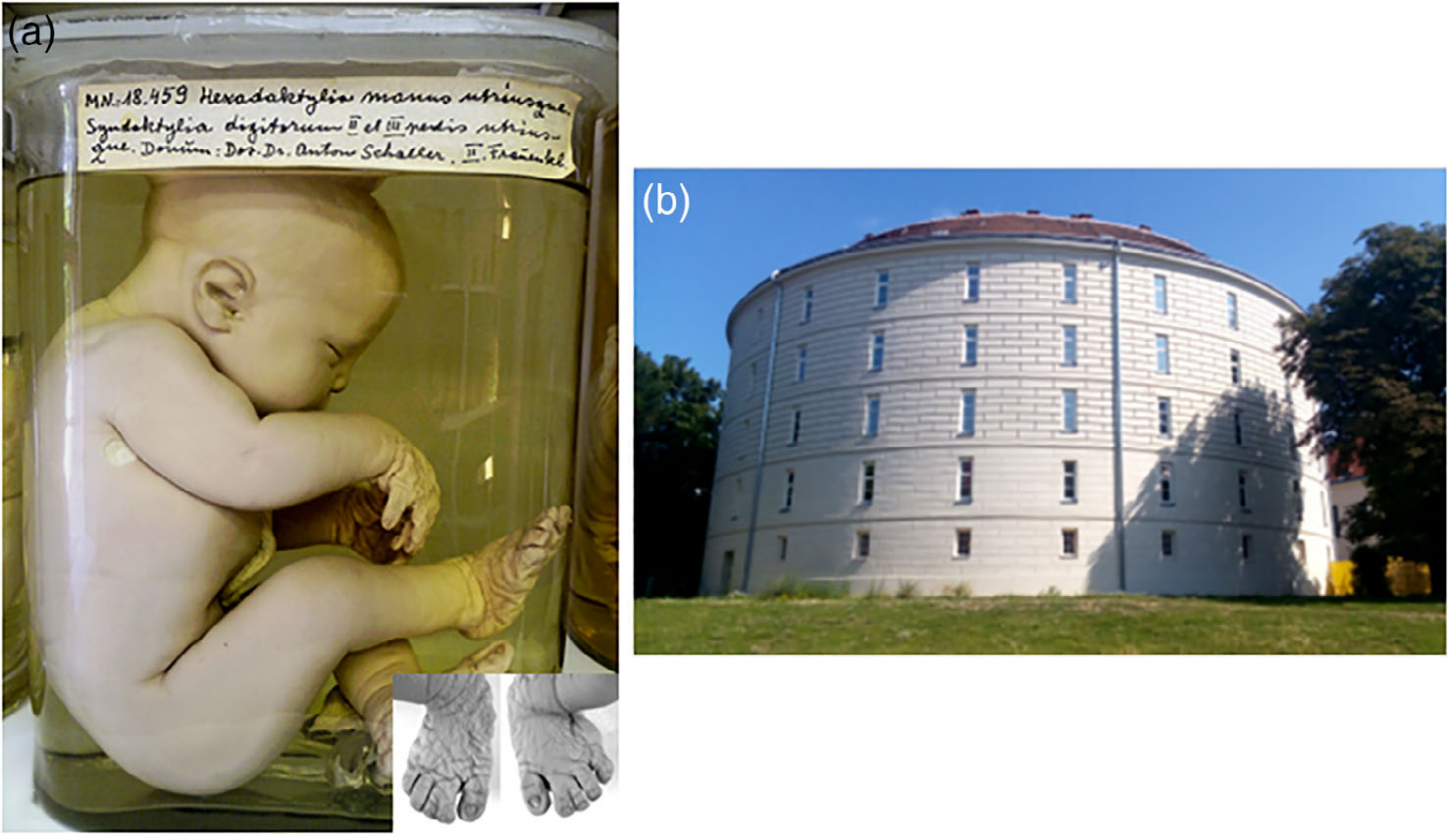
Exhibit from the Fool's tower collection in Vienna with the inscription “hexadaktylia manus utriusque et syndaktylia digitorum II et III pedis utriusque” showing a female preterm infant with facial features of SLOS, postaxial hexadactyly and toe 2/3 syndactyly (a). The Fool's tower (Narrenturm) (b) was constructed in 1784 by Canevale under Emperor Josef II. The tower was central Europe's first equitable accommodation for the mentally ill and composed 139 individual cells. Since 1866 the building has served as a pathological–anatomical museum with more than 20,000 exhibits and wax models (*Campus of the old General Hospital, 1090 Vienna*). SLOS, Smith‐Lemli‐Opitz syndrome

Retrospective SLOS diagnosis in cases 6, 8, and 9 followed an active search for SLOS among “museum specimens” (Figure 3a,b) or among cases with “holoprosencephaly associated with hexadactyly” (Figure 2c,e). Correct syndrome assignment in these cases had been missed at prenatal ultrasound and at autopsy. Cases 8 and 9 had been classified as “pseudotrisomy 13 syndrome” after a normal karyotype was found. These cases represented a “*severe and lethal manifestation*” of SLOS with a severity score of 70. The exhibit of Case 6 (Figure 3a) had a severity score of >35.

### 3.4 Molecular and biochemical analyses

Prenatal or autopsy diagnosis of SLOS was confirmed in Cases 1 to 5, and 7 by molecular genetic studies. Cases 3–5 were homozygous for the splice site mutation c.964‐1G > C (former: IVS8‐1G > C), Case 1 was compound heterozygous for the missense mutations c.976G > T (p.Val326Leu) and c.1054C > T (p.Arg352Trp), Case 2 for c.296 T > C (p.Leu99Pro) and c.1228G > A (p.Gly410Ser), and Case 7 was compound heterozygous for the nonsense mutation c.452G > A (p.Trp151Ter) and the missense mutation c.1351 T > C (p.Cys451Arg) in the *DHCR7* gene (Table 1). Compound heterozygosity was supported by an analysis of paternal DNA which showed heterozygosity for c.1351 T > C only. We were not successful in extracting DNA of sufficient quality from paraffine embedded tissues of Case 6. However, compared to a control case of the same age and fixation, Case 6 exhibited decreased cholesterol values in skin and soft tissues by biochemical cholesterol analysis, which served as confirmation (Table 2). For the siblings of Cases 8 and 9 fetal DNA was no longer available. Retrospective SLOS diagnoses years after autopsy were established by investigation of the parents. The parents were carriers of the *DHCR7* splice site mutation c.964‐1G > C, as was one of their two living sons.

**Table 2:** Cholesterol contents (mg/100 g dry tissue); below: gas chromatography—mass spectrometry graphs from skin samples (left, Case No 6, right, control specimen)

Tissue	Patient/Case No 6	Control case	Ratio (control/Case No 6 ‐ SLOS)
Muscle (abdominal)	0.622232	3.909408	6.28
Adipose tissue	0.066392	0.628408	9.47
Skin	0.080288	0.799792	9.96

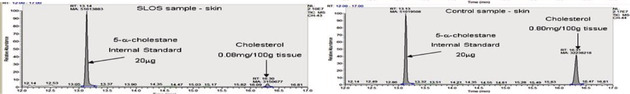

Abbreviation: SLOS, Smith‐Lemli‐Opitz syndrome.

## 4 DISCUSSION

**Figure 4:**
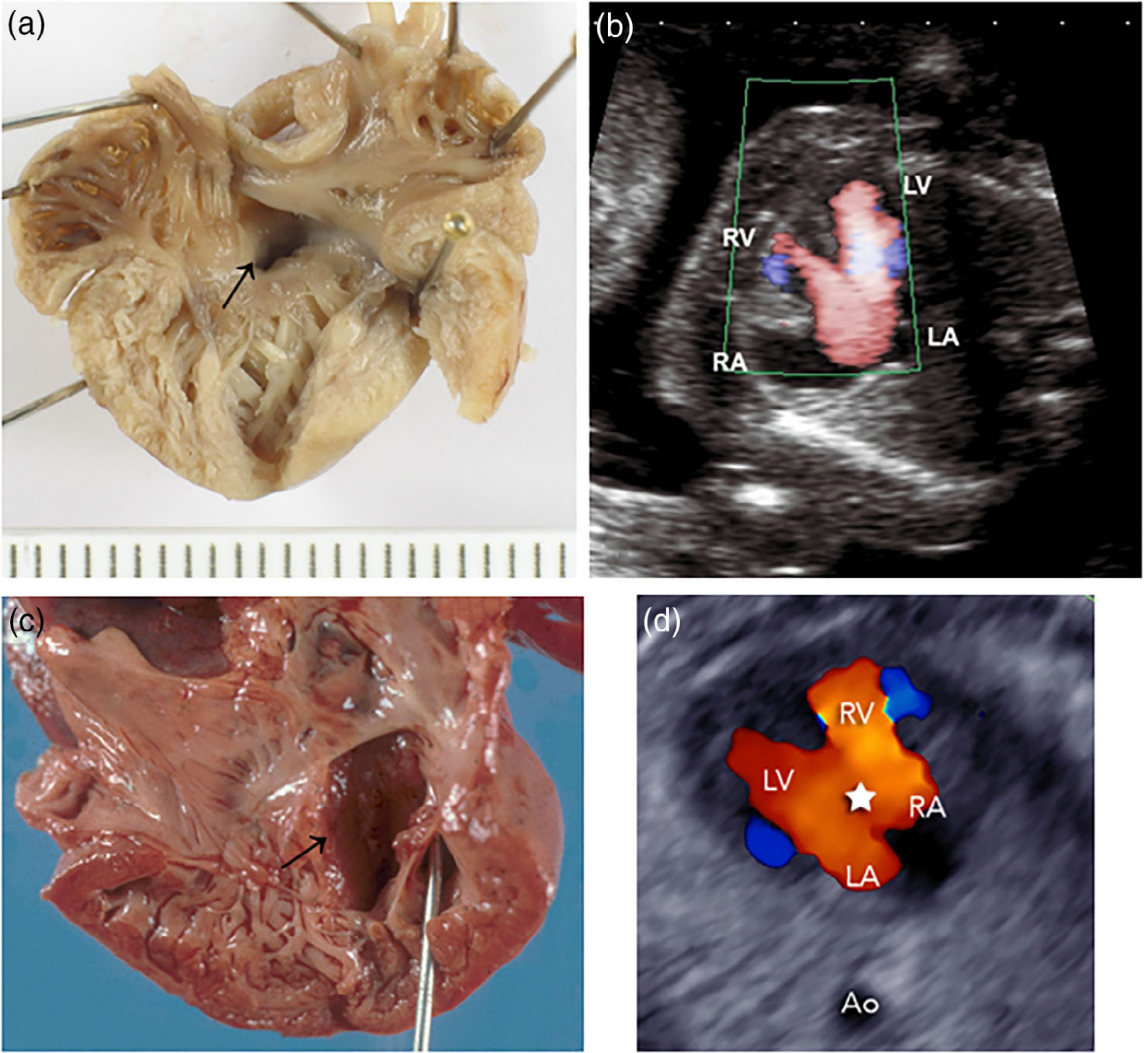
Cardiac inflow tracts of the right ventricles showing partial atrioventricular canal defect (AVSD) with ASD1 (→), minimal upper VSD and tricuspid valves communicating with the mitral valves in Case 7 (a) and complete AVSD with ASD1, large VSD (→) and common atrioventricular valves in Case 9 (c). Corresponding prenatal Doppler sonography of Case 7 (b) and Case 9 (d)

SLOS was suspected at autopsy in four growth retarded fetuses of 18 to 23 gestational weeks (Cases 3–5 and 7) aborted after prenatal ultrasound diagnosis of postaxial hexadactyly, ambiguous genitalia, cardiac defects, renal agenesis, and Dandy‐Walker cyst (Figures 2, 3, 4). Typical facial dysmorphisms, already recognizable in midterm fetuses, and toe II/III syndactyly completed the pattern of characteristic SLOS features (Figure 1). In these fetuses, the suspected pathoanatomical diagnosis was confirmed by biallelic mutations in the *DHCR7* gene, enabling retrospective SLOS diagnosis in two previously undiagnosed siblings of Cases 3 and 4. Two further SLOS fetuses of 12 and 15 gestational weeks (Cases 1 and 2) lacked classical SLOS features; and would have escaped diagnosis at autopsy. Three growth retarded fetuses of 35 to 38 weeks (Cases 6, 8, and 9) were diagnosed retrospectively, years after SLOS diagnosis had been missed at autopsy. The observation of nine primarily undiagnosed cases (seven fetuses and two siblings) among 13 SLOS cases (nine fetuses and four siblings) supports the assumption of a higher than expected incidence of the disorder, if prenatal, often undiagnosed cases are included. This higher incidence had been suggested with regard to the significant discrepancy between the prevalence of the disease (1:22,700) and the inappropriately high rate of mutation carriers (1:51) among Caucasians, indicating a twofold higher prevalence rate of 1:10,404 (Lazarin et al., 2017). Considering the difficulties in recognizing syndrome‐specific facial features in fetuses by prenatal ultrasound or at autopsy by non‐syndromologically oriented pathologists, the syndrome‐specific internal malformation pattern may gain importance for early syndrome recognition.

Forty‐four percent of SLOS patients are born with a cardiovascular malformation. These heart defects include a high rate of atrioventricular septal (= canal) defects (AVSDs = AVCDs) and atrial septal defects (ASDs) and are frequently associated with anomalies of the pulmonary venous return. In fact, the number of AVSDs among 95 SLOS patients with heart defects was 21 (22%) and that of ASD1/ASD2/single atrium was 3/19/1 (24.2%) (Lin, Ardinger, Ardinger Jr., Cunniff, & Kelley, 1997). Fetuses with SLOS show an even higher rate of heart defects, since not all survive until or after birth (Quélin et al., 2012). In our series six of nine fetuses displayed a heart defect, with three corresponding to an AVSD and three to an ASD 1 and/or 2 (Figure 4 a–d). Taken together, the two fetal studies show a heart defect incidence in SLOS fetuses of 79% and of these an AVSD rate of 60%. This finding is similar to the high AVSD rate of 73% among heart defects of fetuses with trisomy 21 compared to a 2.8% AVSD rate among chromosomally normal heart defects in the Baltimore infant study group (Carmi, Boughman, & Ferencz, 1992; Rehder, 1981).

The incidence of renal anomalies in patients with biochemically proven SLOS is 43% (Kelley & Hennekam, 2000). In SLOS fetuses, renal anomalies represent an almost constant feature. The most common features are renal hypoplasia and unilateral renal agenesis. However, bilateral renal agenesis has rarely been described in SLOS, presumably because in the presence of a dominating lethal malformation, the diagnosticians may be misguided to overlook an underlying syndrome. In cases with an affected sibling, diagnostic efforts will focus on a syndromic disorder (Löffler, Trojovsky, Casati, Kroisel, & Uttermann, 2000; Quélin et al., 2012; Stewart, Nevin, & Dornan, 1995).

Major structural brain anomalies have been reported in 37% of biochemically confirmed SLOS patients (Kelley & Hennekam, 2000). Structural brain anomalies in SLOS mainly comprise an absent corpus callosum, enlarged ventricles, hypoplastic cerebellum, and partial or complete absence of the vermis, resulting in diverging cerebellar hemispheres and distension of the remaining roofing membrane of the fourth ventricle to form a Dandy‐Walker cyst (Figure 2 a,b) (Kelley & Hennekam, 2000). HPE with cebocephaly in a biochemically confirmed SLOS patient was first reported in 1996 (Kelley et al., 1996). Meanwhile 12 SLOS cases presenting alobar, semilobar, and lobar HPE have been described. Weaver et al., observed a first case of SLOS with cyclopia. *7DHCR* gene analysis was performed in five of the 11 biochemically proven HPE cases, showing null alleles, deletions and nonsense mutations (Nowaczyk, McCaughey, Whelan, & Porter, 2001; Petracchi, Crespo, Michia, Igarzabal, & Gadow, 2011; Travessa, Dias, Rocha, & Sousa, 2017; Weaver, Solomon, Akin‐Samson, Kelley, & Muenke, 2010). In one SLOS case HPE had developed in the presence of a nonsense and two missense mutations (Quélin et al., 2012).

HPE results from failure of the forebrain to cleave into two hemispheres. In its severest form, “alobar” HPE, it is characterized by a single, small‐sized, horseshoe‐shaped holosphere lacking an interhemispheric fissure at its convexity and by a common ventricle without any midline structures. The common ventricle is covered by leptomeninges at its opening that are continuous with the membranous roof of the third ventricle and are often distended to form a large occipital cyst. In a less severe “semilobar” type the interhemispheric fissure is better developed in the occipital portion, forming a short cleft into the fused frontal forebrain mass (Figure 2d). Even milder “lobar” HPE is characterized by an interhemispheric fissure along the entire midline that shows incomplete division in its depth (Friede, 1975). HPE is often associated with a variety of facial midline defects, leading to hypotelorism and median cleft lip, and palate due to premaxillary agenesis or in its severe forms to cebocephaly (hypotelorism and hypoplastic nose with single nostril), as in our two cases, to ethmocephaly (hypotelorism and interorbital proboscis) or to cyclopia (midline fusion of eyes and supraorbital proboscis) (Friede, 1975; Nanni, Schelper, & Muenke, 2000).

The main differential diagnosis in cases with holoprosencephaly and associated postaxial polydactyly is trisomy 13. In contrast to SLOS fetuses, trisomy 13‐fetuses show large kidneys, rather than renal hypoplasia or agenesis, and truncus anomalies in the cardiac outflow tract rather than a defect in the cardiac inflow tract such as A(V)SD (Rehder, 1982). If a chromosome anomaly is excluded, monogenic HPE syndromes must be also considered. These include Pallister‐Hall syndrome (PHS), as well as HPE9 and Culler‐Jones syndrome (CJS), due to dominant mutations in the *GLI3* and *GLI2* genes, respectively, and hydrolethalus syndrome related to recessive mutations in *HYLS1* and *KIF7* (Kruszka & Muenke, 2018). These disorders are more difficult to delineate from SLOS except for the hypothalamic hamartoma in PHS or the pituitary hypoplasia in HPE9 and CJS. The term “pseudotrisomy 13” is still used for cases, in which the assignment to a monogenic syndrome failed, but will become obsolete with whole exome sequencing.

As previously assumed there is an association between patient's genotype (residual enzyme function) and cholesterol values as well as clinical phenotype (Ciara et al., 2004; Waterham & Hennekam, 2012; Witsch‐Baumgartner et al., 2000). This can also be observed in these fetuses. Case 1 (p.Val326Leu and p.Arg352Trp) and Case 2 (p.Gly410Ser and p.Leu99Pro) carry missense mutations localized in transmembrane domains, and p.Gly410Ser in the fourth cytoplasmic loop of the delta‐7‐sterol reductase. These genotypes are usually associated with a mild to moderate phenotype, confirmed by severity scores of 5 and 15, respectively, in our cases. Cases 3 to 5 and 7 to 9 can be considered more severe or lethal reflected by severity scores of >30 (Kelley & Hennekam, 2000). Five of the more severe cases, including the holoprosencephalic fetuses 8 and 9, carry the homozygous null mutation c.964‐1G > C, whereas Case 7 (severity score of 60) is compound heterozygous for the null mutation p.Trp151Ter and the missense mutation p.Cys451Arg. The p.Cys451Arg is a mutation localized in the putative C‐terminus. A null mutation combined with a C‐terminus mutation has not yet been described, but as such null mutations, compound heterozygous with a transmembrane missense mutation, are associated with more severe phenotypes than homozygosity for transmembrane mutations. Partly depending on the localisation of *DHCR7* missense mutations in different protein domains they may be associated with residual enzyme function and an attenuated clinical phenotype. Thus, it may be assumed that the novel combination of a null allele with a mutation localized in the putative C‐terminus is also associated with a more severe phenotype (Witsch‐Baumgartner et al., 2000).

Our female formalin‐fixed exhibit of the Fool's Tower Collection in Vienna presented with postaxial hexadactyly, syndactyly of toes 2 and 3, ASD2, mild microcephaly and characteristic facial features, allowing the retrospective clinical diagnosis of SLOS (Figure 3a). Due to the long formalin fixation time we did not succeed to obtain DNA of sufficient quality for molecular analysis. However, we were successful in our biochemical investigations that showed up to 10 times lower cholesterol contents in dry muscle, skin and adipose tissues of our SLOS case compared to that in a long‐term formalin‐fixed same age control specimen with a seemingly normal phenotype. In 1997 Oostra et al. (Oostra, Baljet, Schutgens, & Hennekam, 1997) had described a 130‐year‐old SLOS exhibit from the Museum Vrolic Collection of Human Anatomy in Amsterdam. The male specimen showed additional hypospadias. Cholesterol levels in moist and dry skin biopsies were even more reduced with a ratio of 1:45 compared to those in a control specimen with a perioral teratoma. The main advantage of biochemical cholesterol analysis performed by gas chromatography—mass spectrometry is that it can also be applied for the diagnosis of other disorders related to the cholesterol biosynthesis pathway. In the Smith‐Lemli‐Opitz‐like syyndromes, as for example, desmosterolosis and lathosterolosis not only decreased or near normal levels of cholesterol but also elevated levels of its precursors desmosterol and lathosterol, respectively are present in pateint's tissues including skin and internal organs (Kelley, 1995; Kelley & Herman, 2001).

In summary, SLOS remains undiagnosed in many affected fetuses due to variations in fetal phenotypes and lack of experience with fetal syndromology. Classical manifestations should allow a first sight diagnosis at autopsy in the presence of syndrome‐specific facial features, associated with postaxial hexadactyly, syndactyly of Toes II/III and/or hypospadias. For prenatal ultrasound diagnosis the internal malformation in association with postaxial hexadactyly may be of greater diagnostic relevance. These internal malformations include ASDs/AVSDs of the heart, renal anomalies ranging from renal hypoplasia to uni‐ or bilateral renal agenesis and cerebral malformations, for example, Dandy‐Walker cyst and holoprosencephaly. These anomalies are part of a specific malformation pattern and are related to abnormal modification of the SHH morphogen as an effect of decreased cholesterol levels. Differential diagnosis covers a wide spectrum of polydactyly syndromes, but can be restricted under consideration of the internal malformation pattern, especially in cases with HPE.

## Data Availability

Data available on request from the authors
